# Fasciitis-panniculitis syndrome with autoantibodies reacting to adipocyte pericellular fibers: a case report

**DOI:** 10.1186/s12969-025-01071-w

**Published:** 2025-02-18

**Authors:** Yu Uehara, Takuji Enya, Kohei Miyazaki, Yoshiyuki Hakata, Sachiyo Kawahara, Masaaki Miyazawa, Keisuke Sugimoto

**Affiliations:** 1 Department of Pediatrics, Kindai University Faculty of Medicine, 377-2 Ohno-higashi, Osaka-Sayama, 589-8511 Japan; 2 Department of Immunology, Kindai University Faculty of Medicine, Osaka-Sayama, Japan

**Keywords:** Fasciitis-panniculitis syndrome, Autoantibodies, Famotidine, Cutaneous polyarteritis nodosa, Pediatric case

## Abstract

**Background:**

Fasciitis-panniculitis syndrome (FPS) typically presents with swelling and skin hardening. Its histopathological characteristics include inflammatory cell infiltration and fibrous thickening of the subcutaneous tissue and fascia. Panniculitides in children are rare and only a small number of juvenile FPS cases have been reported. We encountered a case of a 10-year-old boy in which autoantibodies reactive to adipocyte pericellular fibers were detected in relapsing FPS.

**Case presentation:**

The patient developed a high fever and skin swelling with pain and erythema on the right side of his body following an abrasion injury on his right wrist at the age of 5 years, and was suspected of having streptococcal toxic shock-like syndrome, for which he received antimicrobials, immunoglobulin therapy, debridement, and plasma exchange. The same manifestations with similar magnetic resonance imaging (MRI) findings of high signal on short tau inversion recovery showing the spread of inflammation in the fat tissue and fascia was observed twice at the age of 6 years. Serological analyses for conventional autoantibodies, bone marrow aspiration, and whole-exome sequencing examination were non-remarkable. Prednisolone was effective in ameliorating the above putative autoinflammatory syndrome. The patient was admitted at the age of 10 years with similar clinical and MRI findings indicative of recurrence of the same disease. En bloc biopsy from the skin to the fascia showed thickening of collagen fibers, infiltration of inflammatory cells composed mainly of neutrophils and lymphocytes, and necrotizing vasculitis in the fat tissue and fascia. Immunohistochemical staining of the en bloc biopsy sections indicated infiltration of T lymphocytes and macrophages in the perivascular connective tissue and fibrinoid necrosis, supporting the diagnosis of FPS. Induction therapy with prednisolone resulted in a remission. IgG purified from the patient’s serum reacted with pericellular basement membranes in the subcutaneous fat tissue by immunohistochemistry. The patient is currently taking famotidine to prevent relapses and is making good progress in his recovery.

**Conclusions:**

Although pathogenic autoantibodies have not been described in FPS, our results suggest that fat-tissue-reactive autoantibodies may be involved in the pathogenesis of FPS.

## Background

Fibrous thickening and chronic inflammation in the subcutaneous septal and fascial collagenous scaffold are the characteristic histologic features of fasciitis-panniculitis syndrome (FPS) along with, in some cases, vasculitis [1–3]. As to the pathogenesis of FPS, it has been suggested that T-lymphocytes recruited by heat shock proteins and chemoattractants are locally activated and release interleukins and interferons. This results in the activation of macrophages, mast cells, and B-lymphocytes, which in turn promotes fibroblast proliferation, collagen synthesis, and vascular endothelial damage [1]. Magnetic resonance imaging (MRI) is useful as an adjunct method to diagnose FPS, while en bloc biopsy that collects a mass of the skin and muscle tissues is necessary for definitive diagnosis. While infection and malignancy are the major causes of secondary FPS, there are idiopathic cases represented by eosinophilic fasciitis (EF) [1]. Although there is no standard treatment, cimetidine [1–3] and prednisolone [4] have been reported to be effective in the treatment of FPS [1–3]. Intravenous cyclophosphamide has also been reported to be effective in prednisolone-refractory cases [5].

In this study, we describe a pediatric case of FPS in which deficiency of adenosine deaminase 2 (DADA2) was ruled out based on whole exome-sequencing and histological findings. Instead, we detected IgG reactive with adipocyte pericellular fibers in patient’s sera.

## Case presentation

**Fig. 1:**
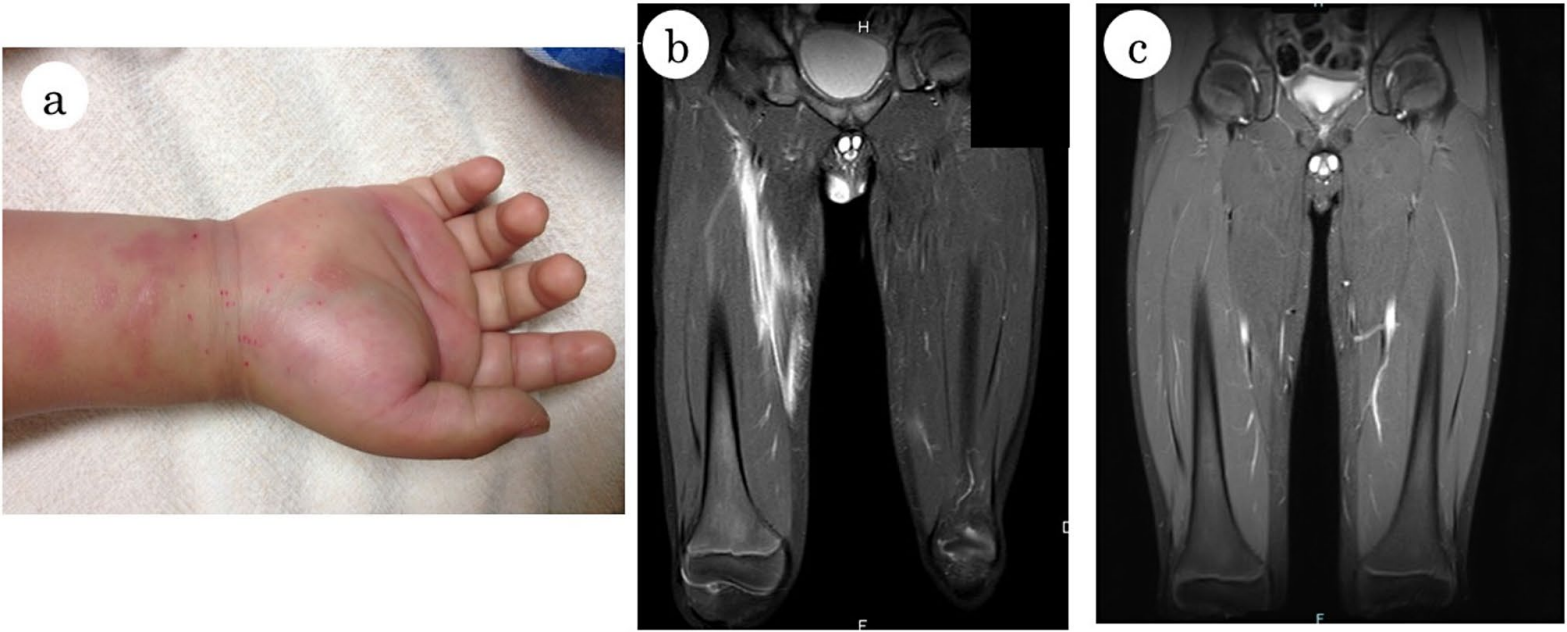
Macroscopic skin manifestations at the initial onset and MRI findings before and after the treatment during recurrence of the present patient. **a**: Skin swelling with erythema at the initial onset on the right upper limb following an abrasion injury on the right wrist. **b**: MRI of the lower extremities showing a high signal area in the right thigh on short tau inversion recovery. **c**: After the treatment, the high signal area in short tau inversion recovery disappeared

The patient was a 10-year-old boy. When he was 5 years old, an abrasion on his right wrist led to progressive swelling of the right upper limb, accompanied by pain and erythema (Fig. 1a). The neck and the right side of the abdomen were also affected. These lesions were accompanied by high fever and pain, elevated anti-streptolysin O (ASO)/anti-streptokinase (ASK) antibodies, and liver dysfunction. MRI of the extremities revealed a marked inflammatory reaction that spread through the fat tissue and fascia. Fulminant streptococcal infection was suspected, and he was treated with antimicrobial agents, immunoglobulin injection, local debridement, and plasma exchange. The same manifestations with similar MRI findings of high signal on short tau inversion recovery showing the spread of inflammation in the fat tissue and fascia were observed twice at the age of 6 years. A search for autoantibodies, bone marrow aspiration, and whole exome sequencing were performed without pathognomonic findings. He was suspected to have an autoinflammatory syndrome and was treated with prednisolone. Subsequently, the patient was kept under outpatient observation without medication. He was 10 years old when he visited our hospital with the chief complaints of fever and difficulty in walking due to pain in the right thigh. Physical examination at the time of admission revealed a height of 149.0 cm (93.5 percentile (%ile)) weight of 38.9 kg (80%ile), body temperature of 38.1℃, blood pressure of 88/49 mmHg, pulse rate of 113 beats per minute. No symmetrical hardening in the skin was observed. There were no abnormalities in the chest and abdomen. There was no notable medical or family history, except for the above preceding events at the ages of 5 and 6 years. Blood tests at the time of the visit showed the following: an elevated white blood cell count, 12,130 /µl (reference range: 4,000–10,700) with neutrophils, 77.6%; lymphocytes, 15.3%; and eosinophils 0.9%; C-reactive protein (CRP), 3.67 mg/dl (reference range: 0–2.57); and serum amyloid A, 818.6 µg/ml (reference range: 0–8). Laboratory test results also revealed an increased blood sedimentation rate, 82 mm/h (reference range: 2–10); aldolase, 10.7 U/l (reference range: 2.1–6.1); and creatinine kinase (CK), 45 U/l (reference range: 51–270). Moreover, aspartate aminotransferase (AST), 47 U/l (reference range: 15–31); alanine aminotransferase (ALT), 54 U/l (reference range: 9–32); fibrinogen, 664 mg/dl (reference range: 200–400); and fibrin degradation products (FDP), 6.5 µg/ml (reference range: 0–0.4) were also elevated.

**Fig. 2:**
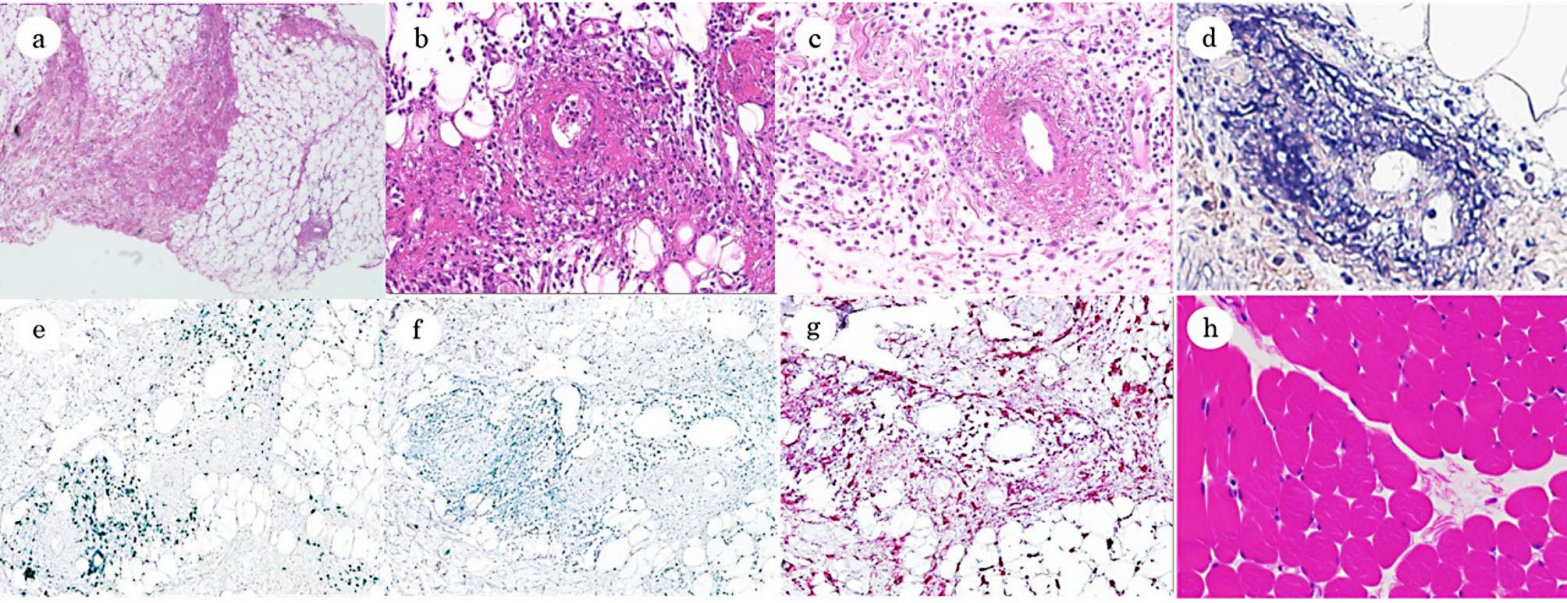
Biopsy findings. **a**: A representative hematoxylin and eosin (HE)-stained section of the patient’s fat tissue showing thickened collagen bundles (×40). **b**: A HE-stained section of the patient’s en bloc biopsy showing infiltration of inflammatory cells into the adipose tissue and necrotizing vasculitis (×100). **c**: A HE-stained section showing edema and diffuse infiltration of inflammatory cells into the perifascia with fibrinoid necrosis of the vascular wall (×100). **d**: A phosphotungstic acid hematoxylin (PTAH)-stained section showing fibrin deposition in the vessel wall (×200). **e**: Immunohistochemical staining showing infiltration of CD3^+^ T lymphocytes into the perivascular area of the adipose septa (×100). Anti-human CD3 rabbit antibody was obtained from Cell Signaling Technology (Danvers, MA, USA). HRP-conjugated anti-rabbit IgG Novolink polymer (Leica Biosystems, Nussloch, Germany), was used as a secondary antibody. **f**: Immunohistochemical staining showing less dominant infiltration of CD19^+^ B lymphocytes into the perivascular area of the adipose septa (×100). Anti-CD19 rabbit antibody was obtained from Cell Signaling Technology and used along with the above Novolink polymer. **g**: Immunohistochemical staining showing infiltration of CD68^+^ macrophages into the perivascular area of the adipose septa (×100). Anti-CD68 antibody [PG-M1] was obtained from abcam. Alkaline phosphatase-conjugated anti-mouse IgG Histofine Simple Stain (Nichirei) was used as a secondary antibody. **h**: A HE-stained section showing unaffected muscle tissue in the same biopsy (×100)

These results indicated an inflammatory response along with elevated aldolase. MRI of the lower extremities showed a high-signal area in the medial right thigh on short tau inversion recovery with the spread of the inflammation to the fat tissue and fascia (Fig. 1b). En bloc biopsy demonstrated thickened collagen bundles in the fat tissue (Fig. 2a), and focal infiltration of inflammatory cells mainly composed of neutrophils and lymphocytes in the fat tissue and fascia along with necrotizing vasculitis (Fig. 2b-d). Immunohistochemical staining revealed infiltration of T lymphocytes and macrophages around the blood vessels (Fig. 2e-g). The muscle tissue was not affected (Fig. 2h). These findings are consistent with the diagnosis of FPS, and prednisolone administration (1 mg/kg/day) was initiated on the day of diagnosis. The dose of prednisolone was reduced to 0.5 mg/kg/day on day 6 and subsequently to 0.38 mg/kg/day on day 9. Thereafter, its dose was further reduced to 0.25 mg/kg/day on day 12 and to 0.13 mg/kg/day on day 13. The next day of treatment initiation, pain in the right thigh disappeared and he was able to walk. The disappearance of the lesions was confirmed by MRI examination of the lower extremities on day 11 (Fig. 1c). On day 14, post-treatment blood test results were as follows: CRP, 0.053 mg/dl; serum amyloid A, 3.9 µg/ml; blood sedimentation rate, 18 mm/h; and aldolase, 5.0 U/l. He was discharged with no worsening of symptoms or exacerbation of clinical data. After his discharge, 0.5 mg/kg/day of famotidine administration was continued to prevent possible flare-ups. The patient was scheduled to see the physician every three months and has not experienced any recurrence or other symptoms for more than two years.

**Fig. 3:**
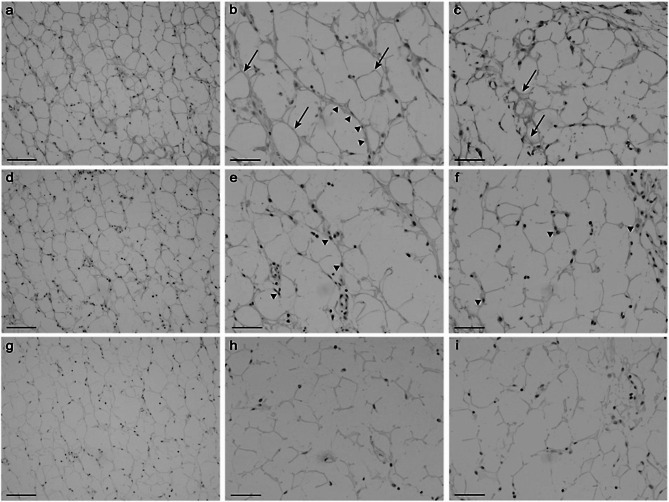
Immunohistochemical staining of the subcutaneous fat tissue using the patient’s IgG. **a**: A representative photomicrograph of the fat tissue stained with biotin-conjugated IgG purified from the patient’s serum. Note diffuse staining of pericellular fibers. Bar = 100 μm. **b** and **c**: Two representative high-power view fields of the fat tissue stained with the biotin-conjugated IgG purified from the patient’s serum. Bar = 50 μm. In panel **b**), the arrows indicate the prominent staining of pericellular fibers, and the arrowheads indicate the staining of fiber bundles. In panel **c**), pericellular staining sometimes shows a membranous pattern. **d**: A representative photomicrograph of the fat tissue stained with biotin-conjugated IgG purified from a control serum. A view field corresponding to that of panel a) is taken. Bar = 100 μm. **e** and **f**: Two representative high-power view fields of the fat tissue stained with the biotin-conjugated IgG purified from the control serum. Bar = 50 μm. Unlike panels **b**) and **c**), no diffuse staining of pericellular fibers is observed. Instead, some nuclei are stained (arrowheads). **g**-**i**: The same fat tissues were incubated with PBS instead of biotinylated IgG. No staining was observed. Bar = 100 μm (**g**) or 50 μm

Serological analyses for conventional autoantibodies including antinuclear antibodies were all negative. Therefore, IgG was purified from the patient’s serum using a previously described method [6] and labelled with biotin to investigate the possible presence of autoantibodies reactive with fat and/or fascial tissues. Control IgG was similarly purified from the serum of a 5-year-old girl who shares the blood group with the patient. The girl presented to the paediatric outpatient clinic with a suspected food allergy and her blood was taken under consent. Equivalent levels of biotin conjugation between the patient’s and control IgG were confirmed by Western blotting.　 Formalin-fixed paraffin-embedded tissue sections derived from the patient’s en block biopsy were processed though hydrated autoclave treatment (121 ℃, 10 min) in 0.01 M citric acid solution for antigen retrieval. Immunohistochemical staining revealed that the IgG derived from the patient’s serum uniformly reacted with pericellular basement membrane (BM)-like structures surrounding each fat cell (Fig. 3a-c). At the same concentration (0.09 ng/ml), biotin-conjugated control IgG showed no uniform staining of pericellular BM, although some nuclei were stained (Fig. 3d-f). As adipocyte extracellular matrix shift from fibrillar to BM collagens during adipogenic differentiation [7] and collagen type IV is the major collagen of BM [8], we next asked if the patient’s IgG reacts with human collagen type IV using available enzyme-linked immunosorbent assays (ELISA). As a control, Antibodies specific to collagen type I were detected using collagen type I-coated microtiter plates, an avidin-HRP conjugate, and 3,3’,5,5’-tetramethyl-[1,1’-biphenyl]-4,4’-diamine (TMB) substrate, all provided in the Human Collagen Type I ELISA kit (ACEL Inc. Sagamihara, Japan). Antibodies specific to collagen type IV were detected using microtiter plates pre-coated with an antibody specific for collagen type IV, a collagen type IV solution, an avidin-HRP conjugate, and a chemiluminescent substrate, all provided in the Human Collagen IV ELISA Kit (NOVUS BIOLOGICALS, Centennial, CO, USA). No specific reaction of the patient’s IgG to either type of human collagen was detected (data not shown), indicating that the patient’s IgG reacts with a BM component(s) other than type IV collagen.

## Discussion and conclusions

We experienced a pediatric case of FPS with frequent recurrence, and immunohistochemical and ELISA analyses suggested that an autoantibody or autoantibodies reactive with an adipocyte BM component other than type IV collagen is present in the patient’s serum. Although it is unclear if the detected fat-tissue-reactive autoantibodies are a cause or result of the tissue damage, they may be involved in the pathogenesis of this disease.

Since the age of 5 years, this patient had experienced four episodes of skin swelling, with the MRI findings demonstrating the spread of inflammation to the fat tissue and fascia, which was consistent with the observed skin pathology. En bloc biopsy of the skin and fascia showed thickening of collagenous fibers in the fat tissue, infiltration of inflammatory cells consisting mainly of neutrophils and lymphocytes from the fat tissue to the fascia, as well as necrotizing vasculitis, leading to the diagnosis of FPS. From a clinical point of view, systemic sclerosis should be a diagnosis of exclusion, and from a pathological point of view, DADA2 and otulipenia should be considered as possible causes [9]. Raynaud’s phenomenon, anti-Scl-70, anti-centromere, and anti-U1-RNP autoantibodies were all negative in the patient, which are atypical for systemic sclerosis along with the pediatric onset. DADA2 has been reported to be one of the causes of cutaneous polyarteritis nodosa (CPN) [9, 10] and we did observe necrotizing arteritis in the patient’s biopsy specimen. DADA2 is an autosomal recessive genetic disorder caused by a heterozygous missense mutation of the *ADA2* gene, which decreases the enzymatic activity of adenosine deaminase 2, a protein necessary for the differentiation of vascular endothelial cells. This deficiency results in vascular damage, which may lead to juvenile-onset vasculitis and stroke. An association between DADA2 and CPN has been reported [9–12]. CPN is characterized by necrotizing vasculitis involving all layers of the vessel wall with fibrinoid degeneration of the arteries, and there have been cases of its conversion to systemic polyarteritis nodosa [13–15]. In fact, a case of DADA2 has been reported in which the patient was diagnosed with CPN at the age of 4 years and had systemic symptoms such as fever, headache, fatigue, and vomiting at the age of 13 years, leading to whole-exome sequencing [10].

Otulipenia is an autosomal recessive autoimmune disease caused by mutations in the gene encoding OTULIN, a ubiquitin kinase involved in the NF-κB signal transduction pathway [9]. This mutation causes constant activation of the NF-κB signalling in fibroblasts and mononuclear cells, resulting in symptoms such as fever, arthralgia, and lymphadenopathy due to a cytokine storm [16, 17]. The main pathological changes of oulipenia are observed in medium-sized vessels [9]. In the present case, the characteristic skin manifestations of CPN were clinically absent, collagen fibers in the fat tissue were increased and thickened, and there was the infiltration of inflammatory cells from the fat tissue to the fascia, thus excluding CPN from the possible diagnoses. Furthermore, as no obvious abnormality was found in the whole-exome sequencing, vasculitis caused by DADA2 or otulipenia was excluded.

The pathogenesis of FPS is thought to involve the release of cytokines by T lymphocytes that are activated by heat shock proteins and chemoattractants at the site of injury, resulting in macrophage activation, fibroblast proliferation, and vascular endothelial damage. Although there is no established treatment for FPS, the efficacy of cimetidine [1–3], prednisolone [4], and cyclophosphamide [5] has been reported. Due to frequent relapses in this patient, famotidine was selected, and no subsequent relapse was observed. The mechanism of the therapeutic effect of cimetidine on FPS has not been clarified, but it has been reported that cimetidine efficiently modifies T cell functions and thereby exerts immunomodulatory effects [2, 18, 19]. Immunohistochemical staining of the specimens prepared from the en block biopsy revealed infiltration of T lymphocytes and macrophages in the perivascular connective tissue and lesions of necrotizing vasculitis; consequently, prednisolone was selected for initial treatments. We expected that prednisolone might suppress cytokine-mediated activation of the macrophages as well as fibrosis of the fascia and fat tissue.

Comparing our pediatric case to adult cases of FPS, differences in skin manifestations might be present. Naschitz et al. demonstrated that sleeve-like and/or plaque-like lesions were observed in all 32 adult cases [1]. Furthermore, some adult cases showed skin ulcers [1, 5]. Our patient, on the other hand, primarily presented with skin swelling accompanied by pain and, in some instances, erythema. Interestingly, however, no differences were observed between the adult and pediatric cases in histopathologic and MRI findings. Serological findings of FPS are generally characterized by negative antinuclear antibodies and other known autoantibodies. Thus, autoantibodies possibly involved in the pathogenesis of FPS have not been described. However, in our case, IgG purified from the patient’s serum uniformly reacted with pericellular BM structures of the adipose tissue. Clinically, antibodies to denatured human type IV collagen are common in patients with autoimmune diseases such as rheumatoid arthritis, scleroderma and systemic lupus erythematosus [20]. However, IgG purified from the patient’s serum did not react with human collagen type I or IV, suggesting that autoantibodies targeting BM components other than type IV collagen may be involved in the pathogenesis of FPS in our case.

In the present case, frequently recurring panniculitis and fasciitis were observed. When initial symptoms such as high fever, pain and swelling of the extremities and erythema are observed in a patient and FPS is suspected, MRI can be performed as an adjunct diagnostic modality. However, en bloc biopsy from the subcutaneous to the muscle tissue should be performed for a definitive diagnosis and confirmation of the areas that are infiltrated with inflammatory cells. Therefore, we believe that en bloc biopsy is crucial for the diagnosis of FPS. Whether the currently detected autoantibodies reactive with adipocyte BM are actually involved in the pathogenesis of FPS needs to be investigated in the future by accumulating more cases.

## Data Availability

No datasets were generated or analysed during the current study.

## Abbreviations

ALT: Alanine aminotransferase
ASK: Anti-streptokinase antibody
ASO: Anti-streptolysin O antibody
AST: Aspartate aminotransferase
BM: Basement membrane
CK: Creatinine kinase
CPN: Cutaneous polyarteritis nodosa
CRP: C-reactive protein
DADA2: Deficiency of adenosine deaminase 2
EF: Eosinophilic fasciitis
ELISA: Enzyme-linked immunosorbent assays
FDP: Fibrin degradation products
FPS: Fasciitis-panniculitis syndrome
MRI: Magnetic resonance imaging
TMB: 3,3’,5,5’-tetramethyl-[1,1’-biphenyl]-4,4’-diamine
